# IL-4 prevents adenosine-mediated immunoregulation by inhibiting CD39 expression

**DOI:** 10.1172/jci.insight.157509

**Published:** 2022-06-22

**Authors:** Fengqin Fang, Wenqiang Cao, Yunmei Mu, Hirohisa Okuyama, Lingjie Li, Jingtao Qiu, Cornelia M. Weyand, Jörg J. Goronzy

**Affiliations:** 1 Division of Immunology and Rheumatology, Department of Medicine, Stanford University, Stanford, California, USA.; 2 Department of Medicine, Palo Alto Veterans Administration Healthcare System, Palo Alto, California, USA.; 3 Department of Laboratory Medicine, Tongren Hospital, Shanghai Jiao Tong University School of Medicine, Shanghai, China.; 4 Department of Immunology, Mayo Clinic College of Medicine and Science, Rochester, Minnesota, USA.; 5 Health Sciences Institute, China Medical University, Shenyang, China.; 6 Department of Histoembryology, Genetics and Developmental Biology, Shanghai Jiao Tong University School of Medicine, Shanghai Key Laboratory of Reproductive Medicine, Shanghai, China.; 7 Department of Medicine/Rheumatology, Mayo Clinic College of Medicine and Science, Rochester, Minnesota, USA.

**Keywords:** Aging, Adaptive immunity

## Abstract

The ectonucleotidase CD39 functions as a checkpoint in purinergic signaling on effector T cells. By depleting eATP and initiating the generation of adenosine, it impairs memory cell development and contributes to T cell exhaustion, thereby causing defective tumor immunity and deficient T cell responses in older adults who have increased CD39 expression. Tuning enzymatic activity of CD39 and targeting the transcriptional regulation of *ENTPD1* can be used to modulate purinergic signaling. Here, we describe that STAT6 phosphorylation downstream of IL-4 signaling represses CD39 expression on activated T cells by inducing a transcription factor network including GATA3, GFI1, and YY1. GATA3 suppresses *ENTPD1* transcription through prevention of RUNX3 recruitment to the *ENTPD1* promoter. Conversely, pharmacological STAT6 inhibition decreases T cell effector functions via increased CD39 expression, resulting in the defective signaling of P2X receptors by ATP and stimulation of A2A receptors by adenosine. Our studies suggest that inhibiting the STAT6 pathway to increase CD39 expression has the potential to treat autoimmune disease while stimulation of the pathway could improve T cell immunity.

## Graphical abstract

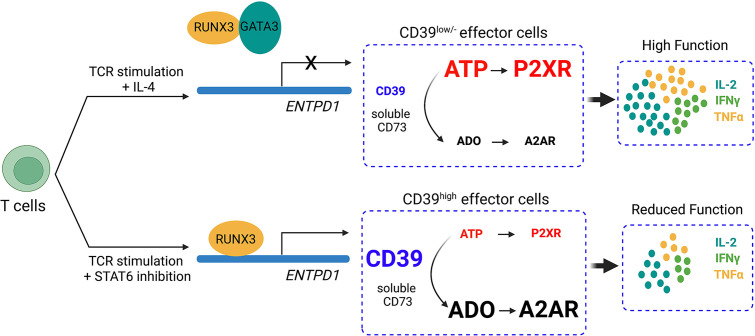

## Introduction

Purinergic signaling is an important mechanism in fine tuning immune cell functions (1–3). Extracellular ATP, released by damaged and dying cells, is a danger signal that plays a proinflammatory function. In T cell differentiation, ATP is secreted by activated T cells and stimulates P2X receptors (P2RX) (4). Calcium influx through P2RX7 has been shown to be pivotal for generating long-lived memory and tissue-resident effector CD8 T cells (5). P2RX4 is recruited to the T cell receptor (TCR) synapse, provides costimulatory signals, and regulates migration (6). CD39 and CD73 are the 2 most critical molecules with enzymatic activities to calibrate the purinergic signals. CD39 depletes secreted ATP, thereby inhibiting P2RX stimulation. The cooperation between CD39 and CD73 produces extracellular adenosine that acts as a mostly immunosuppressive signal to modulate the functions of immune cells. In addition, extracellular AMP produced by CD39 in the absence of CD73 is also immunosuppressive through binding to P1 receptors (7). Taken together, CD39 expression on the cell surface largely determines whether the microenvironment is proinflammatory or immunoregulatory (1–3).

CD73 is constitutively expressed on most naive CD8 T cells and 20%–40% of naive CD4 T cells (8). Upon activation, it is downregulated but re-expressed on a smaller fraction of CD4 and CD8 central and effector memory T cells (8, 9). In contrast, CD39 expression is inducible in naive and memory T cells by TCR stimulation. CD39^+^CD4^+^ T cells have functional characteristics of effector T cells with increased apoptosis susceptibility, at least in part due to stimulation of the A2A receptor (A2AR) by adenosine (10). They have a reduced potential to differentiate into long-term memory T cells. T cells from older adults are more prone to CD39 expression due to more sustained mTORC1 signaling, which may explain their defect in establishing long-lived memory T cells (10, 11). CD39 expression also accounts for defective T follicular helper (TFH) cell differentiation in older age; and stimulation of the A2AR activates the cAMP/PKA/pCREB pathway, which in turn suppresses BCL6 function (12). Moreover, Gupta et al. found CD39 to be coexpressed with PD-1 on effector T cells in chronic lymphocytic choriomeningitis virus (LCMV) infection but not in acute infection. They proposed CD39 to be a T cell exhaustion marker (13). Canale et al. described tumor-infiltrating CD8^+^ T cells with high expression of CD39 that were absent in lymphoid organs but increased in frequencies with tumor growth (14). Taken together, CD39 expression on T cells and the associated depletion of ATP and increased production of adenosine contribute to T cell dysfunction in immune aging and antitumor responses. Therefore, targeting this pathway is an opportunity to enhance immunity, and several forms of intervening with purinergic signaling, in part in combination with checkpoint inhibition, are being explored (15, 16).

While interfering with adenosine signaling appears currently more feasible, targeting CD39 directly has the advantage of maintaining extracellular ATP (eATP) concentrations that are important for the differentiation of memory T cells (5). An alternative to pharmacological inhibition of the enzyme activity is to prevent CD39 expression in T cells. Several studies have explored the transcriptional control of *ENTPD1*. On endothelial cells, hypoxia-induced Sp1 upregulates CD39 expression (17). Liao et al. found that the cAMP/PKA/pCREB pathway mediated the transcriptional regulation of CD39 expression on murine macrophages (18). This later pathway is also pertinent for T cells, where adenosine generated by CD39/CD73 augments *ENTPD1* transcription in a positive feedback loop (12). IL-6–activated pSTAT3 and TGF-β–driven downregulation of GFI1 promoted CD39 and CD73 expression on murine TH17 cells (19). BCL6 can essentially abrogate CD39 expression through directly binding to the *ENTPD1* promoter, consistent with the observation that TFH cells do not express CD39. In contrast, RUNX3, essential for effector cell differentiation, transcriptionally upregulated CD39 expression on T cells (12). Therefore, transcription factor networks quintessential for T cell differentiation and largely driven by cytokines drive CD39 expression.

To identify actionable cytokine-transcription factor networks, we screened cytokines for their ability to influence CD39 expression induced by TCR stimulation and found that IL-4 inhibited *ENTPD1* transcription through the phosphorylation of STAT6 and the upregulation of GATA3, GFI1, and YY1. GATA3 functioned by interacting with RUNX3 and blocking RUNX3 binding to the *ENTPD1* promoter. The ability of STAT6 inhibition to upregulate CD39 expression may be useful for treating autoimmune disease, while activation of the pathway in an immune response has the potential to prevent T cell dysfunction.

## Results

### IL-4 signaling inhibits CD39 expression.

**Figure 1:**
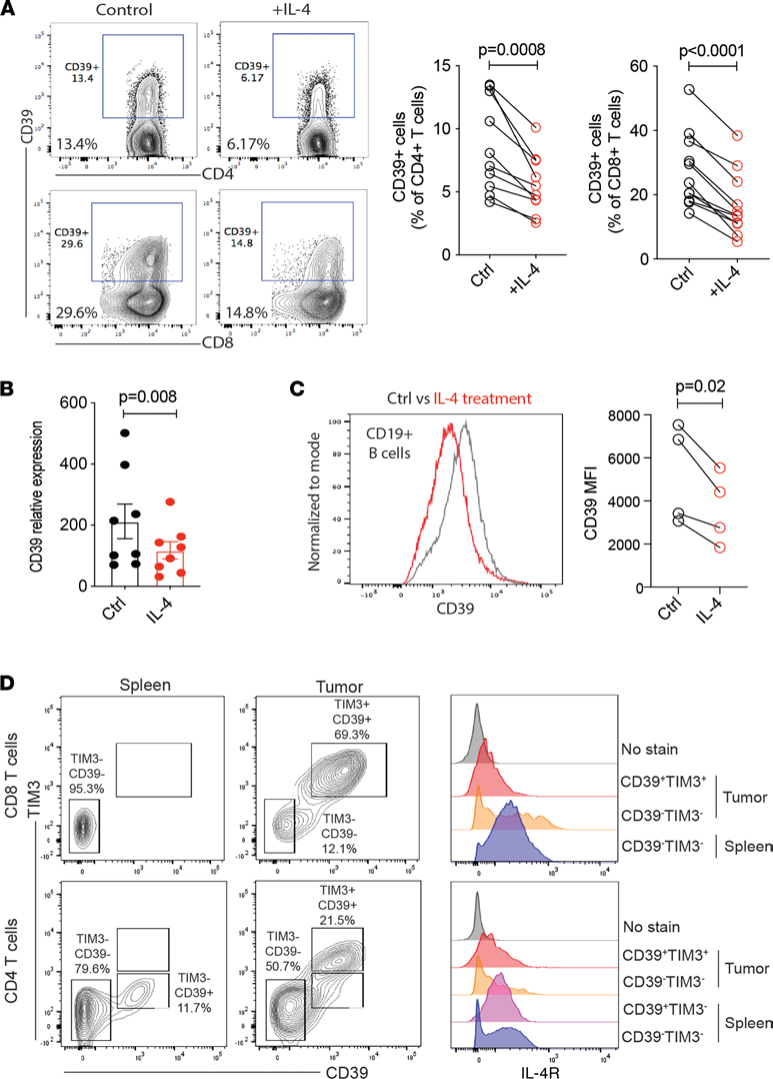
IL-4–induced STAT6 signaling inhibits CD39 expression. (**A**) Purified total T cells from human peripheral blood were activated for 4 days by anti-CD3/CD28 Dynabeads in the presence or absence of IL-4 (20 ng/mL). CD39 expression was compared by flow cytometric analysis. Left are representative contour plots and right are summary results. (**B**) Total T cells were treated as described in **A** and collected for *ENTPD1* transcript quantification. (**C**) PBMCs were activated for 4 days with soluble anti-CD3 Ab (2 μg/mL) in the presence or absence of IL-4. Results on CD39 expression on gated CD19^+^ B cells are shown as representative histograms (left) and summary data (right). (**D**) Mice were implanted with B16 melanoma cells. Splenocytes and TILs were harvested on day 26 and expression of IL-4R on indicated CD4 and CD8 T cell subsets was determined. Contour plots of CD39 and TIM3 expression (left) and histograms of IL-4R expression (right) are representative of 2 experiments. Data in **B** are shown as mean ± SEM and compared by 2-tailed paired Student’s *t* test in **A**–**C**.

CD39 is a checkpoint in purinergic signaling that is induced in selected subsets of effector T cells. The preferential induction on TH1 and TH17 cells and the virtual absence on TFH cells (12, 19) suggest the importance of the initial cytokine milieu for the transcriptional regulation of *ENTPD1*. To identify cytokines that prevent expression, we activated total T cells in the presence of IL-2, IL-4, IL-7, IL-15, TNFα, Type I IFN, and Type II IFN. IL-4 was the only cytokine that downregulated CD39 expression (Supplemental Figure 1A; supplemental material available online with this article; https://doi.org/10.1172/jci.insight.157509DS1). It did so in both naive and memory T cells, suggesting that the effect was not limited to TH2 differentiation (Supplemental Figure 1B). Subsequent experiments in a larger cohort confirmed inhibition of CD39 expression on both CD4 and CD8 T cells by IL-4 (Figure 1A). Inhibition occurred at the level of *ENTPD1* transcription (Figure 1B) and was not limited to T cells but was also found upon treating B cells with IL-4 (Figure 1C). The finding on B cells demonstrated that IL-4 not only prevented the induction but also decreased the constitutive expression of CD39. However, exhausted T cells had downregulated the IL-4R, suggesting that they are no longer susceptible to the IL-4 effect. We analyzed splenic and tumor-infiltrating T cells (TILs) from B16 melanoma/Ova-bearing mice (Figure 1D). Most TIL-expressing PD-1 and TIM3 coexpressed CD39. These cells had reduced IL-4R expression compared with CD39^-^ CD4^+^ and CD8^+^ splenic T cells. In contrast to TILs, splenic CD39^+^ CD4^+^ T cells lacking TIM3, likely resembling Tregs, had normal IL-4R.

### STAT6 inhibition in a T cell response upregulates CD39 expression.

**Figure 2:**
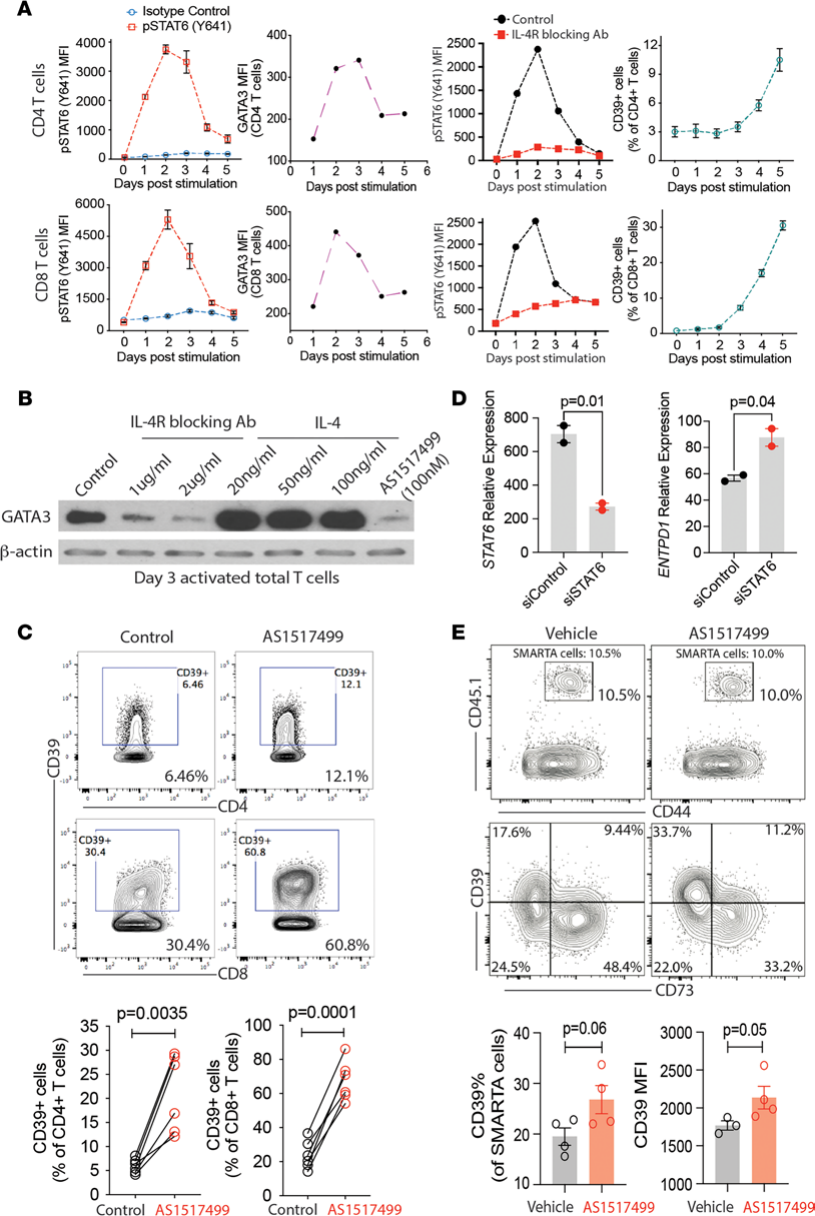
STAT6 inhibition in a T cell response upregulates CD39 expression. (**A**) Purified total T cells were activated for 5 days by anti-CD3/CD28 Dynabeads. Each day, cells were examined for the expression of pSTAT6, GATA3, and CD39 in CD4 or CD8 T cells by flow cytometry. In addition, the level of pSTAT6 was also compared in T cells with or without IL-4 receptor blocking Abs. (**B**) Purified total T cells were activated for 3 days by anti-CD3/CD28 Dynabeads in the presence or absence of IL-4 (20, 50, or 100 ng/mL), IL-4 receptor blocking Abs (1 or 2 μg/mL), or STAT6 inhibitor AS1517499 (100 nM) as indicated. GATA3 expression was assessed by Western blotting. (**C**) Purified total T cells were activated in the presence or absence of the STAT6 inhibitor AS1517499 (100 nM). After 4 days, CD39 expression was evaluated by flow cytometry. (**D**) Total T cells were activated for 2 days before nucleofection with STAT6-specific siRNA smartpool. After 2 additional days, cells were collected for *ENTPD1* and *STAT6* mRNA quantification by real-time qPCR. (**E**) CD45.1^+^ naive SMARTA cells were isolated and injected into CD45.2 B6 WT mice via tail vein, followed by LCMV Armstrong infection and daily i.p. injection of the STAT6 inhibitor AS1517499 or DMSO solvent control. After 6 days, SMARTA cells were isolated from spleen and examined for CD39 expression using flow cytometry. Representative contour plots (upper panel) and summary data (lower panel). Data in **D** are shown as mean ± SEM and compared by 2-tailed paired or unpaired Student’s *t* test in **C** and **E**.

IL-4R stimulation results in STAT6 phosphorylation. In cultures of purified total T cells activated by anti-CD3/CD28 Dynabeads, we observed STAT6 (Y641) phosphorylation starting on day 1 and subsiding on day 4 after stimulation (Figure 2A). In parallel, GATA3 was upregulated upon activation and downregulated on day 4 after stimulation (Figure 2A). This initial peak in STAT6 phosphorylation was inhibited by blocking the IL-4R (Figure 2A), suggesting autocrine or paracrine activity of IL-4. CD39 expression started to rise on day 3 and then progressively increased, suggesting that the delay in the expression of CD39 was due to the transient production of IL-4 in these cultures (Figure 2A). Western blotting confirmed the expression of GATA3 for 3 days after activation of T cells under nonpolarizing conditions (Figure 2B). GATA3 expression was inhibited by adding IL-4R blocking Abs to the culture; the addition of IL-4 served as a positive control (Figure 2B). AS1517499 is a potent and selective STAT6 inhibitor through inhibition of STAT6 phosphorylation and blocking pSTAT6 binding to DNA sequence (20–22). Inhibition of GATA3 expression was seen even at the low concentration of 100 nM (Figure 2B).

Culture of T cells purified from human peripheral blood with AS1517499 for 4 days induced CD39 expression on a markedly higher fraction of CD4 and CD8 T cells compared with control-treated cells (Figure 2C). Furthermore, transfecting T cells with STAT6-specific siRNA SMARTPOOL increased *ENTPD1* transcription (Figure 2D). To examine whether STAT6 inhibition upregulated CD39 expression on T cells in vivo, we used the LCMV infection mouse model. Naive CD45.1^+^ SMARTA CD4 T cells, specific for LCMV GP61-80, were adoptively transferred into CD45.2^+^ B6 WT mice, followed by LCMV infection and daily AS1517499 treatment for 6 days. Flow cytometric analysis showed increased CD39 expression on SMARTA cells in STAT6 inhibitor-treated mice compared with the control group (Figure 2E). Taken together, pSTAT6 suppresses activation-induced CD39 expression in T cells.

### GATA3-mediated inhibition of ENTPD1 transcription is independent of DNA binding.

**Figure 3:**
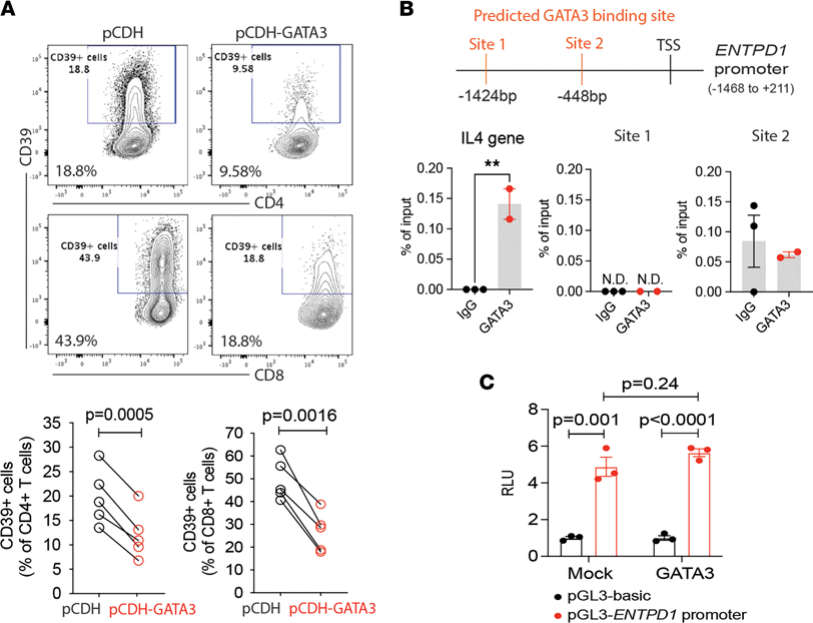
GATA3 suppresses CD39 expression. (**A**) T cells were transduced with GFP-expressing lentivirus, carrying the pCDH or pCDH-GATA3 construct. After 5 days following activation, CD39 expression was evaluated on GFP^+^ T cells by flow cytometry. Representative contour plots and summary data are shown. (**B**) 2 GATA3 binding sites are predicted in the *ENTPD1* promoter region (–1468 to +211 from TSS) by MatInspector. Purified total T cells were activated for 3 days and collected for ChIP using anti-GATA3 Abs. Real-time qPCR was done on 2 predicted sites. The GATA3-binding site in the *IL-4* gene was detected as a positive control. Data are shown as percentage of input. (**C**) HEK293 T cells were transfected with pcDNA3.1-GATA3 and pGL3-*ENTPD1* promoter plasmids. pcDNA3.1 and pGL3-basic were used as control vectors. Renilla luciferase reporter plasmid was taken as a control for transfection efficiency. After 48 hours, luciferase activities were determined. Results are pooled from 3 experiments. Data are shown as mean ± SEM and compared by 2-tailed paired or unpaired Student’s *t* test in **A** and **C**. **P < 0.005.

Lentiviral transduction of human T cells with GATA3 significantly reduced CD39 expression after activation on both CD4 and CD8 T cells (Figure 3A). We predicted 2 GATA3 binding sites in the CD39 promoter sequence at –448 bp and –1424 bp relative to transcription start site (TSS) using public online software Matinspector. However, we did not detect GATA3 binding to these sequences in ChIP assays of T cells activated for 3 days, in contrast to the *IL4* gene promoter, which served as a positive control (Figure 3B). GATA3 ChIP-seq data on in vitro differentiated human TH1 and TH2 cells from the public resource of Cistrome (23) did not yield any peaks in the entire *ENTPD1* gene while peaks in the *IL4* gene region proved the quality of the data set (Supplemental Figure 2). Luciferase reporter assays provided further evidence that GATA3 does not directly regulate the *ENTPD1* promoter. Compared with controls, GATA3 overexpression in HEK293T cells did not reduce the luciferase intensity regulated by the *ENTPD1* promoter (Figure 3C). Taken together, transcription factor GATA3 downstream of IL-4 signaling indirectly suppresses CD39 expression.

### GATA3 reduces ENTPD1 expression through inhibiting RUNX3.

**Figure 4:**
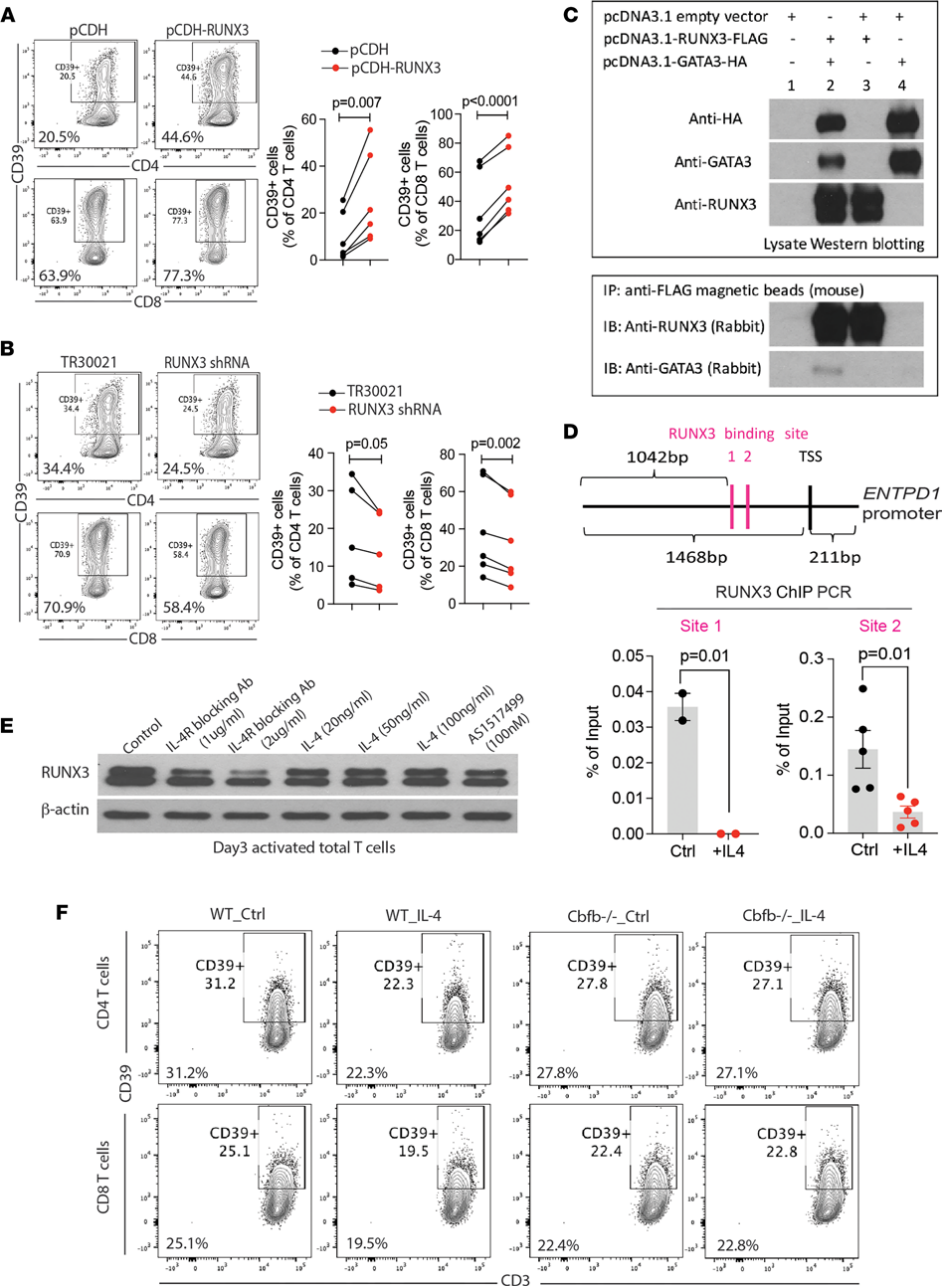
GATA3 prevents RUNX3 binding to the *ENTPD1* promoter. (**A** and **B**) T cells were transduced with GFP-expressing lentivirus, expressing pCDH-RUNX3 or pCDH (control vector), RUNX3-shRNA, or TR30021 (control vector) to overexpress or knockdown RUNX3 during T cell activation. After 5 days, CD39 expression on GFP^+^ T cells was determined by flow cytometry. Representative contour plots and summary data are shown. (**C**) HEK293 T cells were transfected with pcDNA3.1-RUNX3-FLAG, pcDNA3.1-GATA3-HA, or both. pcDNA3.1 was used as a control vector. After 48 hours, cells were collected for CoIP assay using anti-FLAG magnetic beads to pulldown RUNX3. Western blots of total lysates (upper panel) and anti-FLAG pull downs (lower panel) are shown. (**D**) Purified total T cells were activated for 4 days by anti-CD3/CD28 Dynabeads in the presence or absence of IL-4. Cells were collected for RUNX3 ChIP, followed by qPCR of 2 RUNX3 binding sites in the *ENTPD1* promoter. Data are shown as a percentage of input. (**E**) Purified total T cells were activated by anti-CD3/CD28 Dynabeads under indicated conditions and collected on day 3 for Western blotting of RUNX3. (**F**) Splenocytes were isolated from WT and Cbfb^–/–^ B6 mice and cultured on plates precoated with anti-CD3 and CD28 Abs (both 8 μg/mL) in the presence or absence of IL-4 (20 ng/mL). After 4 days, CD39 expression was compared by flow cytometry. Results are representative of 2 experiments. Data in **D** are shown as mean ± SEM and compared by 2-tailed paired or unpaired Student’s *t* test in **A**, **B**, and **D**.

Several groups have described the physical interaction between GATA3 and RUNX3 to block each other’s binding to DNA sequences (24, 25). RUNX3 is a positive regulator of CD39 expression on T cells through binding to the *ENTPD1* promoter (12). Lentiviral overexpression of RUNX3 during T cell activation significantly upregulated CD39 expression on CD4 and CD8 T cells (Figure 4A). Conversely, RUNX3 knockdown using a commercial shRNA lentivector plasmid reduced CD39 induction compared with the control group (Figure 4B). To determine whether GATA3 inhibits CD39 expression through preventing RUNX3 binding to the *ENTPD1* promoter, we first performed CoIP assays of tagged RUNX3 and GATA3 overexpressed in HEK293T cells. When using anti-FLAG magnetic beads to pull down RUNX3, a weak but clear GATA3 band was detected, confirming the physical interaction between RUNX3 and GATA3 (Figure 4C). Previous work identified RUNX3 binding to 2 sites in the *ENTPD1* promoter (12). When ChIP assays were performed with T cells activated for 4 days in the presence and absence of IL-4, a significant suppression of RUNX3 binding to these 2 sites was observed with IL-4 (Figure 4D). The reduced binding was not due to reduced RUNX3 expression. We collected T cells activated for 3 days in the presence of IL-4, STAT6 inhibitor, and IL-4R blocking Abs, respectively. High doses of IL-4R blocking Abs may slightly reduce, but certainly did not increase, RUNX3 expression. The other treatments had no effect (Figure 4E). To further prove that the inhibition of IL-4R signaling on CD39 expression depends on the presence of RUNX3, we activated splenocytes from WT and *Cbfb*^–/–^ B6 mice for 4 days with plate-coated anti-CD3/CD28 Ab in the presence or absence of IL-4. IL-4 reduced CD39 expression on CD4 and CD8 T cells from WT but not in *Cbfb*-deficient mice (Figure 4F).

### IL-4–induced transcription factor network repressing ENTPD1 includes GFI1 and YY1.

**Figure 5:**
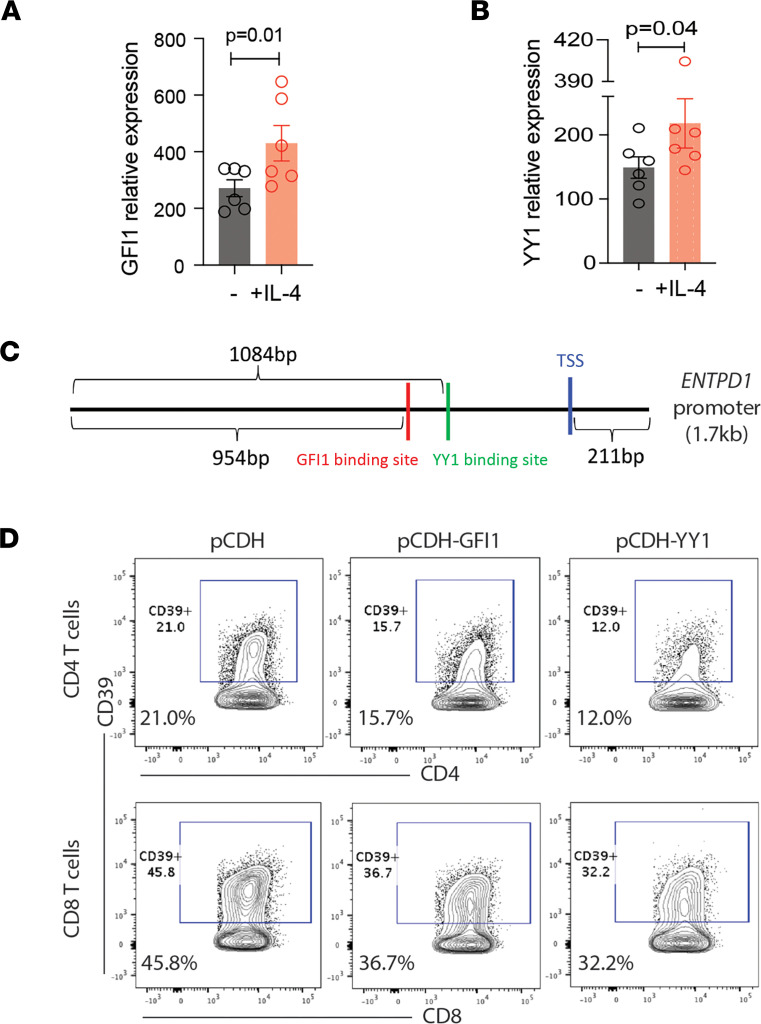
IL-4–induced GFI1 and YY1 repress CD39 expression. (**A** and **B**) Purified total T cells were activated for 4 days with anti-CD3/CD28 Dynabeads in the presence or absence of IL-4. GFI1 and YY1 transcripts were quantified by qPCR. Data were compared by paired 2-tailed Student’s *t* test. (**C**) GFI1 and YY1 binding sites were predicted for the *ENTPD1* promoter sequence (–1468 to +211bp from TSS) using MatInspector. (**D**) Full-length GFI1 and YY1 cDNA were cloned into the pCDH lentivector carrying a GFP reporter gene. Purified total T cells were activated and transduced with the lentiviral constructs. After 5 days, CD39 expression on GFP^+^ T cells was analyzed by flow cytometry. The contour plots are representative of 2 experiments.

In addition to GATA3, transcription factors GFI1 and YY1 were also induced by IL-4 (Figure 5, A and B). Binding sites of GFI1 and YY1 are both predicted in the *ENTPD1* promoter sequence (Figure 5C). GFI1 is a transcriptional repressor that has been shown to suppress CD39 expression in mouse Th17 cells (19). YY1 can be a transcriptional repressor or activator; its activity is reduced in T cells from older adults, which are more prone to CD39 expression (10, 26, 27). We cloned the full length of human GFI1 and YY1 cDNA into pCDH lentivectors containing a GFP reporter gene. Lentiviral overexpression of GFI1 or YY1 in activated human T cells significantly reduced CD39 induction (Figure 5D).

### STAT6 inhibition suppresses T cell function through CD39 upregulation.

**Figure 6:**
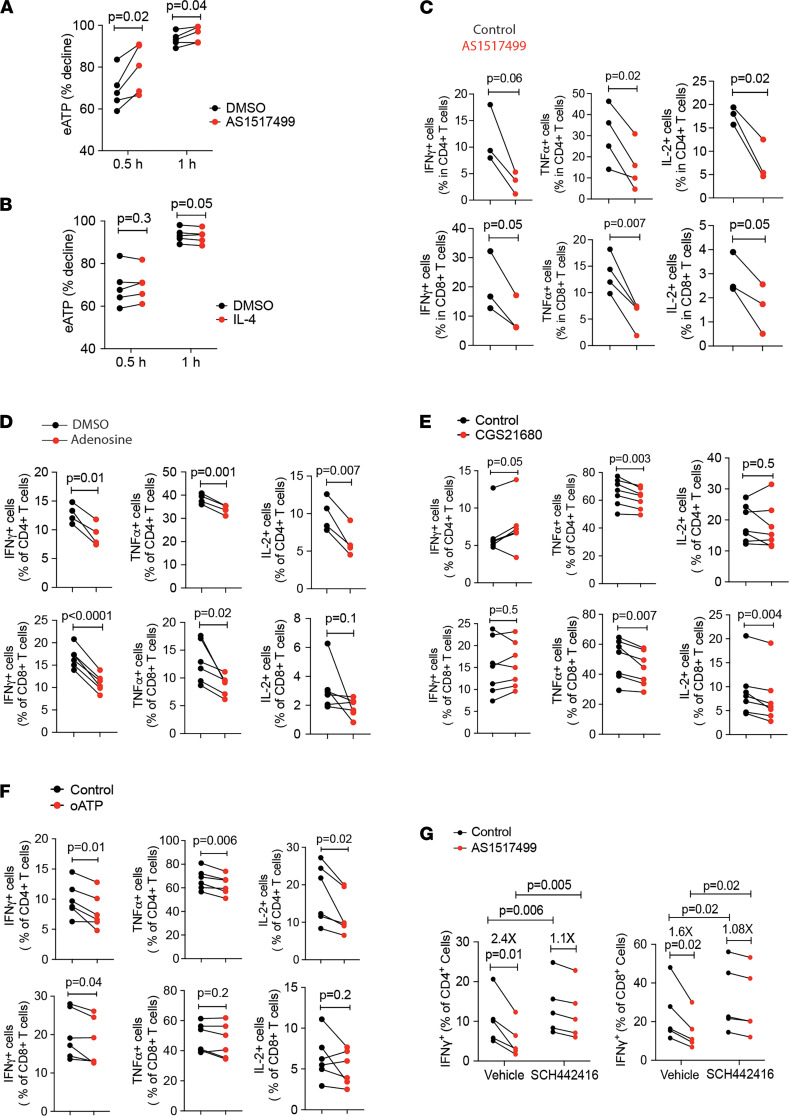
STAT6 inhibition suppresses T cell function through CD39 upregulation. (**A** and **B**) T cells were stimulated in the absence or presence of the STAT6 inhibitor AS1517499 (100 nM for **A**) or 20 ng/mL IL-4 for B. Cells were resuspended in medium supplemented with ATP at 20 μM on day 4 and eATP concentrations were determined after 30 minutes and 1 hour. Results are shown as a percent decline from original concentration. (**C**) Purified T cells were activated for 4 days in the absence or presence of AS1517499, then restimulated with newly added Dynabeads for 6 hours before cells were collected for intracellular cytokine staining and flow cytometry analysis. (**D**–**F**) Day 4-activated T cells were restimulated with newly added Dynabeads plus/minus adenosine (500 μM for **D**), or the A2AR agonist CGS21680 (100 nM for E), or the P2RX antagonist oATP (100 μM for F) for 6 hours and then stained for intracellular cytokines. (**G**) Purified T cells were treated with STAT6 inhibitor AS1517499, A2AR antagonist SCH58621, or both during T cell activation by Dynabeads. On day 4, cells were restimulated by PMA/IONO for 4 hours before being collected for intracellular cytokine staining. Results are pooled from 3 experiments. Data in **C** are shown as mean ± SEM and compared by paired 2-tailed Student’s *t* test in **A**–**G**.

CD39 on activated effector T cells functions as a checkpoint of purinergic signaling by depleting eATP and initiating the generation of adenosine that binds to P1 receptors. eATP can be detected in day 4 culture of T cells after activation, but not in medium in the absence of cells, suggesting active secretion of ATP (Supplemental Figure 3). To determine whether the increased expression of CD39 upon STAT6 inhibition is sufficient to change eATP, we added 20 μM ATP to day 4-T cell cultures and monitored eATP over the subsequent hour. T cells with the AG or GG genotype of the *ENTPD1* SNP at rs_10748643 that allows for CD39 expression on effector T cells were used. Cells activated in the presence of 100 nM STAT6 inhibitor AS1517499 for 4 days were more efficient at degrading ATP than control cultures (Figure 6A). We had 1 sample from an adult with the A/A genotype which fails induction of CD39 expression. Here, no effect of the STAT6 inhibitor on eATP was detected (Supplemental Figure 3). The relative changes in CD39 expression on day 4 after IL-4 treatment were small (Figure 1). The addition of 20 ng/mL IL-4 to the cultures had no effect on eATP depletion after 30 minutes and had a small but significant effect at 1 hour (Figure 6B).

To determine whether the increased expression of CD39 has functional consequences, we assessed cytokine production upon restimulation of T cells that were activated in the presence of the STAT6 inhibitor and found reduced generation of IL-2, IFN-γ, and TNFα (Figure 6C). STAT6 is known to regulate other transcription factors, such as IRF4 (28), BLIMP1 (29), and TCF1 (30) that could affect the ability to produce cytokines independent of CD39. We therefore performed experiment to determine whether the decline is due to purinergic signaling. Restimulation of activated T cells by anti-CD3/CD28 Dynabeads in the presence of 500 μM adenosine for 6 hours reduced production of cytokines in CD4 and CD8 T cells including IFN-γ, TNFα, and IL-2 (Figure 6D) suggesting that generation of adenosine may account for the defective effector function of these T cells; however, required adenosine concentrations were high. Results using the A2AR selective agonist CGS21680 at 100 nM in the same system showed consistent inhibition for TNFα on CD4 and CD8 T cells but less so for IL-2 and not for IFN-γ (Figure 6E). Conversely, restimulation of cells in the presence of the P2RX inhibitor oxidized ATP (oATP) also suppressed cytokine production upon restimulation, suggesting that the defective effector function could be directly caused by eATP depletion (Figure 6F). To directly address the question whether the inhibition of cytokine secretion by the STAT6 inhibitor was through upregulated CD39 expression and generation of adenosine from endogenous ATP, purified T cells were treated with STAT6 inhibitor (AS1517499), A2AR antagonist (SCH442416), or a combination of both during T cell activation. Addition of the A2AR inhibitor improved cytokine production in both settings, but even more so in effector T cells that were generated in the presence of the STAT6 inhibitor. The inhibitory effect of STAT6 inhibition was nearly completely abrogated by A2AR blocking (Figure 6G). These data support the notion that the inhibition of T cell function by AS1517499 was mainly mediated by elevated CD39 expression and adenosine production, although a contribution of additional eATP depletion by CD39 is possible.

## Discussion

The ectonucleotidase CD39 is the rate-limiting enzyme in the degradation of extracellular ATP. As a major checkpoint in purinergic signaling, it is a target for immune intervention; inhibiting its enzymatic activity or preventing its expression on activated T effector cells is immunostimulatory and would therefore be beneficial in particular settings of T cell exhaustion. Here, we describe transcription factors downstream of IL-4 function as repressors of *ENTPD1*, thereby improving T cell responses upon restimulation through preventing adenosine production or ATP depletion. IL-4-induced GATA3 inhibited *ENTPD1* transcription indirectly by physically interacting with RUNX3, blocking its recruitment to the *ENTPD1* promoter. GFI1 and YY1, both also TFs induced by IL-4, reduced CD39 expression when overexpressed in T cells. Conversely, STAT6 inhibition greatly increased the frequency of T cells that expressed CD39 in an immune response, indicating its application in the treatment of autoimmune diseases.

In recent years, several strategies have been explored to manipulate the ATP-adenosine pathway to improve immune responses to tumors. CD73 antagonists and inhibitors of A2A receptors have shown efficacy in improving tumor immunity. More recent studies have employed inhibitors of CD39 that have the advantage of not only inhibiting the generation of adenosine but also the degradation of ATP, thereby preserving ATP’s proinflammatory and immune stimulatory activity (16, 31). An alternative approach to pharmacological inhibition of CD39 is to prevent its expression. CD39-deficient mice generated by genetic KO of the *Entpd1* gene exhibited dysregulation of many physiological processes (32, 33), consistent with the wide tissue distribution of CD39. Moreover, human siblings with a stop-codon mutation in *ENTPD1* developed neurological and gastrointestinal complications (34). These data suggest that significant side effects must be expected from global CD39 repression or inhibition. However, human adults with a frequent SNP at the position rs10748643 of the *ENTPD1* gene are largely healthy with the exception of an increased susceptibility to inflammatory bowel disease (35). The SNP is responsible for different levels of CD39 expression in T cells. Individuals with the GG variant express high CD39 on T cells upon activation, while those with the AA allele induce CD39 only on a very small number of T cells and only with a low cell surface density. Consequently, individuals with an AA allele are able to generate more TFH cells (12) and have better vaccine responses, while in those with the GG allele, effector T cells are more short-lived (10). Interestingly, the effect of the allelic polymorphisms is mainly limited to T cells and not seen with B cells or monocytes, suggesting cell-type specific transcriptional regulation, which, therefore, opens the opportunity for selective targeting that appears to be safe.

Several transcription factors have been implicated in regulating *ENTPD1* transcription. Initial studies have been spearheaded by Pinsky’s group, mostly in cell types that have constitutive expression of CD39. Hypoxia was shown to induce CD39 expression through the transcription factor SP1 (17). Regional expression on endothelial cells was in part determined by KLF2 under laminar shear stress (36). pCREB regulated the *ENTPD1* promoter in mouse macrophages (18). Also, several studies have examined the transcriptional control of *ENTPD1* in T cells. pCREB, also important in T cells, is a possible target for intervention. Inhibition of cyclooxygenase and prostaglandin synthesis impaired pCREB-dependent transcription of *ENTPD1*. pCREB is also part of an important feedback loop downstream of adenosine-mediated A2AR stimulation, thereby susceptible to A2AR blockers (12). In CD8 T cells, CD39 expression is regulated by NAD(P)H oxidases, possibly through an ROS-dependent activation of NF-κB and JNK pathways (37). In vitro generation of murine TH17 cells is associated with the expression of CD39 and CD73 through IL6-mediated STAT3 signaling and TGF-β–mediated downregulation of Gfi-1 (19); however CD39 and CD73 are rarely coexpressed in human T cells and the vast majority of human CD4^+^ CD39^+^ effector T cells are not TH17 cells. A key transcription factor in CD4 T cells is BCL6. Loss of BCL6 or gain in the antagonistic transcription factor BLIMP1 is required for CD39 to be expressed on effector T cells (12). Increased expression of BLIMP1, unleashing *ENTPD1* transcription in T cells from older adults appears to be 1 mechanism that curtails effector T cell survival and impairs their function in older age (10, 11).

An earlier study has found reduced CD39 expression in TH2 cell-driven diseases, indicating a role of the IL-4 signaling pathway in *ENTPD1* transcription. *ENTPD1* transcripts were significantly lower in PBMC of patients with asthmatic diseases compared with healthy controls and were inversely correlated with IL-4 and GATA3, but positively correlated with TGF-β and FOXP3 transcripts (38). Here, we show that IL-4 is indeed actively repressing *ENTPD1* by inducing GATA3, GFI1, and YY1. This effect was seen irrespective of TH2 differentiation; in fact, studies were done on total T cells and, therefore, mostly lineage-committed T cells that continued to produce IFN-γ and TNF upon restimulation. GATA3 functions by competing with the activity of RUNX3 that is absolutely required for the induction of CD39 expression after T cell activation. The IL-4 signaling pathway, therefore, emerges as a promising druggable target to manipulate purinergic signaling in effector T cells. Moreover, the amount of IL-4 produced at the initiation of an immune response may have downstream effects through attenuating CD39 expression, preventing this arm of T cell exhaustion. Lymphocytes mainly express the type I IL-4 receptor, which is formed by IL-4Rα with the γc chain. The binding affinity of IL-4 to IL-4Rα is very high; therefore IL-4 at a very low concentration can maximally occupy the receptor chains (39). In our polyclonal activation assay, stimulation of total T cells under TH0 conditions in the absence of exogenous IL-4 induced phosphorylation of STAT6, likely due to the activation of infrequent IL-4–producing cells. Elevated pSTAT6 during T cell activation under nonpolarizing conditions has been shown to play an important role in T cell differentiation, such as the downregulation of TCF1 (30) and maintaining IRF4 (28) and FOXP3 (40, 41) expression. Our experiments in the LCMV infection model showed that STAT6 inhibition upregulated the expression of CD39 on LCMV-specific SMARTA cells, providing additional evidence that the IL-4-STAT6-GATA3 pathway is activated and relevant in antiviral responses in vivo. Stimulation with IL-4 during the first days of T cell activation could therefore prevent CD39 expression that contributes to exhaustion. A previous study has shown that CD39 inhibition restored cytokine production, consistent with our findings, but that PD-1 inhibition induced proliferation (42). Thus, different interventions may be needed to improve functionality of exhausted T cells. It should be noted that expression of IL-4R was low on exhausted TIM3^+^CD8 T cells, rendering them resistant. IL-4, therefore, can only be used to prevent, not inhibit, CD39 expression on exhausted cells.

While the major focus of targeting CD39 is on improving or preventing T cell exhaustion in antitumor responses or optimizing vaccine response in older adults, its immunosuppressive function is likely beneficial in the setting of autoimmune disease. The rs10748643 G/G SNP causing high expression on T cells is associated with reduced production of effector cytokines IL-17 and IFN-γ (43), reduced graft versus host disease in a human mouse chimera model (44), and reduced generation of TFH cells (12). The AA SNP associated with low CD39 expression was linked to increased susceptibility to Crohn’s disease (35). *ENTPD1* KO mice have a high susceptibility to murine colitis (35). It is undetermined whether this effect is related to the absence of CD39 on professional Tregs, but upregulation of CD39 on effector T cells may curtail pathological inflammatory T cell responses. We found that the potent and selective STAT6 inhibitor AS1517499 is a powerful inducer of CD39 expression on in vitro cultured CD4 and CD8 T cells (Figure 2A). In vivo experiments also showed upregulation in antigen-specific CD4 T cells during acute LCMV infection (Figure 2E). T cells treated with AS1517499 had decreased cytokine production in CD4 and CD8 T cells, including IL-2, IFN-γ, and TNF, that was likely a combined effect of ATP depletion and reduced P2RX stimulation plus adenosine generation and A2AR stimulation. While a direct effect of STAT6 inhibition on T cell differentiation, for example by a failure of inducing IRF4 (45), cannot be excluded, the reversibility by the A2AR antagonist SCH442416 implicates the increased CD39 expression. In addition to potential applications of STAT6 inhibitors in TH2-driven diseases, they may also be useful to prevent excessive inflammatory responses in viral infection or treat autoimmune disease by increasing adenosine signaling.

## Methods

### Study population and cell purification.

Deidentified leukocyte buffy coats or leukocyte reduction chambers from 78 donors were purchased from the Stanford University Blood Bank and the Mayo Clinic Blood Bank. Untouched total T cells were purified using a RosetteSep Human T cell enrichment cocktail (STEMCELL Technologies, catalog 15061). PBMCs were obtained by density gradient centrifugation using Lymphoprep (STEMCELL Technologies, catalog 07861). Purity of isolated cells was greater than 90%.

### Cell culture and flow cytometry.

Lymphocytes were cultured in RPMI-1640 (Sigma, catalog R8758) with 10% FBS and antibiotics. The medium itself, in the absence of cells, did not have any detectable ATP. Cells were activated for 4 days by anti-CD3/CD28 Dynabeads (Thermo Fisher Scientific, catalog 11132D; bead to cell ratio 1:2) in the presence or absence of cytokines or chemical compounds, including STAT6 inhibitor AS1517499 (100 nM; Axon Medchem, catalog Axon 1992), IL-4 (20 ng/mL; Peprotech, catalog 200-04), and A2AR antagonist SCH442416 (10 μM; TOCRIS, catalog 2463). In the initial screening assay, the following cytokines and concentrations were used to treat cells during T cell activation: IL-2, 200 units/mL; IL-4, 40 ng/mL; IL-7, 40 ng/mL; IL-15, 40 ng/mL; TNF, 4000 units/mL; type I IFN, 5000 units/mL; and type II IFN, 100 ng/mL (all from Peprotech). For in vitro Th0, Th1, and Th2 differentiation/plasticity assays, purified naive or memory CD4 T cells were activated and cultured with 20 ng/mL human recombinant IL-12 (Peprotech) and 5 μg/mL anti–IL-4 (eBiosciences) for Th1 polarization; and with 10 ng/mL IL-4 and 10 μg/mL anti–IFN-γ (BioLegend) for Th2 polarization. For siRNA knockdown experiments, cells were activated with Dynabeads for 2 days; beads were then removed, and cells were nucleofected/electroporated with STAT6-specific siRNA SMARTPOOL (Dharmacon, catalog M-006690-01-0005). After 2 additional days, cells were collected for *ENTPD1* and *STAT6* mRNA quantification by real-time quantitative PCR (qPCR).

For cell surface staining, cells were incubated with fluorescence-conjugated Abs in FACS buffer (5% FBS in PBS) for 30 minutes at 4°C. The following Abs and reagents were used for human T cell surface staining: anti-CD3 Ab (OKT3, BioLegend, catalog 317326); anti-CD4 Ab (RPA-T4, BioLegend, catalog 300506); anti-CD8 Ab (RPA-T8, BioLegend, catalog 301016); anti-CD39 (A1, BioLegend, catalog 328206); anti-CD19 Ab (4G7, BioLegend, catalog 392506); and Live/dead Fixable Aqua dead cell stain kit (Thermo Fisher Scientific, catalog L34966). To examine pSTAT6 or GATA3 kinetics, cells were activated for 5 days by anti-CD3/CD28 Dynabeads with or without IL-4R blocking Abs (R&D Systems, catalog MAB230). Cells were collected on each day for surface staining and fixation (BD Biosciences, catalog 554655). Permeabilization (Perm buffer III, BD Biosciences, catalog 558050) and staining with PE anti-pSTAT6 Tyr641 (BioLegend, catalog 686003) or APC GATA3 Ab (BioLegend, catalog 653806) were performed on all fixed samples in parallel at the end of the longitudinal experiments. For intracellular cytokine staining, activated cells were restimulated with fresh anti-CD3/CD28 Dynabeads (1:1 ratio) for 6 hours in the presence or absence of adenosine (500 μM), the P2RX antagonist oATP (100 μM, MilliporeSigma, CAS 71997-40-5), or the A2AR agonist CGS21680 (100 nM); or with PMA/IONO (phorbol 12-myristate 13-acetate, 50 ng/mL; Peprotech, catalog 1652981; and Ionomycin, 500 ng/mL; Peprotech, catalog 5608212) for 4 hours in the presence of Brefeldin A (GolgiPlug) at 37°C. The cells were sequentially processed with surface staining, fixation/permeabilization with Cytofix/Cytoperm plus kit (BD Biosciences, catalog 555028), followed by final staining with fluorescence-labeled Abs specific to the indicated cytokines, including anti–IFN-γ Ab (B27, BD Biosciences, catalog 562016), anti-TNF Ab (MAb11, BioLegend, catalog 502916), and anti-IL-2 Ab (MQ1-17H12, BioLegend, catalog 500322).

For cell surface staining of mouse T cells, spleen cells isolated from WT or CD2cre^+^Cbfb^fl/fl^ B6 mice were activated for 4 days by plate-coated anti-CD3/CD28 Abs (8 μg/mL each, eBiosciences) in the presence or absence of mouse IL-4 (20 ng/mL; Peprotech, catalog 214-14). CD39 expression on T cells was analyzed by flow cytometry. The following Abs were used: anti-CD3 (145-2C11, Thermo Fisher Scientific); anti-CD4 (RM4-5, BioLegend); anti-CD8 (53-6.7, BioLegend), and anti-CD39 (Duha59, BioLegend). In addition, anti-CD73 (TY/23, BD Biosciences); anti-CD44 (IM7, BioLegend); anti-CD45.1 (A20, BioLegend); and anti-CD45.2 (104, BioLegend) were used for the in vivo experiments.

Cells were analyzed on an LSRII or LSR Fortessa (BD Biosciences), and flow cytometry data were analyzed using FlowJo (TreeStar).

### Quantification of extracellular ATP.

Purified total T cells were activated with anti-CD3/CD28 Dynabeads for 4 days. eATP in the supernatant was quantified using the ATP determination kit (Thermo Fisher Scientific). Briefly, supernatants were incubated with the premade mix including luciferase and its substrate luciferin for 10 minutes at room temperature. Luminescence was read using BioTek Synergy H1. ATP concentrations were calculated from a standard curve.

Purified T cells from the donors with AG or GG genotype at rs10748643 were stimulated in the presence or absence of AS1517499 (100 nM) or IL-4 (20 ng/mL). On day 4, cells were resuspended in medium supplemented with 20 μM ATP. ATP concentrations in the supernatant were determined after incubation for 30 minutes and 1 hour.

### Cell lines.

HEK293T cells (ATCC) were grown in DMEM supplemented with 10% FBS and 100 U/mL penicillin and streptomycin (Thermo Fisher Scientific). B16-OVA cells were grown in complete DMEM medium supplemented with 400 μg/mL G418 (Thermo Fisher Scientific).

### Plasmids construction, lentivirus production, and transduction.

Lentiviral transduction was used to overexpress GATA3 and RUNX3 in human T cells. Human *GATA3* or *RUNX3* was inserted into lentivector (pCDH-GFP-Em-CD513B-1, System Biosciences) using the following primers: hRUNX3_enzy_F: 5′ AACTAGCTAGCatggcatcgaacagcatcttcg 3′ and hRUNX3_enzy_R: 5′ ATACGCGGATCCtcagtagggccgccacac 3′; and hGATA3_enzy_F: 5′ AACTAGCTAGCatggaggtgacggcgga 3′ and hGATA3_enzy_R: 5′ ATAAGAATGCGGCCGCctaacccatggcggtgacc 3′. *shRUNX3* lentiviral vector (Origene, catalog TL309682) was used to silence *RUNX3* in human T cells. Lentivirus was produced by transfection of the lentiviral vector, along with psPAX2 and pMD2.G (Plasmid 12260 and 12259, Addgene) into HEK293T cells using Lipofectamine LTX (Thermo Fisher Scientific, catalog 15338100). Lentiviral particles were collected 48 and 72 hours after transfection, filtered through a 0.45 mm syringe filter (MilliporeSigma), concentrated using Peg-it solution (System Biosciences) and titered on HEK293T cells. For lentiviral transduction, fresh human total T cells were activated with anti-CD3/CD28 Dynabeads and transduced with lentivirus produced from HEK293T cell in the presence of 8 mg/mL polybrene (MilliporeSigma) and 10 U/mL human IL-2 (Peprotech). After 48 hours, the medium was changed, and cells were cultured for a total of 4 days before downstream analysis.

### Western blotting.

Cells were lysed in RIPA buffer containing PMSF and protease and phosphatase inhibitors (Santa Cruz Biotechnology) for 30 minutes on ice. Proteins were separated on denaturing 4%–15% SDS-PAGE (Bio-Rad), transferred onto PVDF membrane (MilliporeSigma), and probed with Abs to GATA3 (D13C9), RUNX3 (D6E2), and β-actin (13E5, Cell Signaling Technology). Membranes were developed using HRP-conjugated secondary Abs and Pierce ECL Western blotting substrate (Thermo Fisher Scientific).

### CoIP assay.

Human *GATA3* or *RUNX3* was inserted into pcDNA3.1 vector by PCR-based amplification. The cloning primers were as follows: RUNX3-FLAG F: 5′ AACTAGCTAGCATGGACTACAAAGACGATGACGACAAG atggcatcgaacagcatcttcg 3′; RUNX3-FLAG R: 5′ TTTGGCGCGCC tcagtagggccgccacac 3′; GATA3-HA F: 5′ AACTAGCTAGCATGTACCCATACGATGTTCCAGATTACGCT atggaggtgacggcgga 3′; and GATA3-HA R: 5′ TTTGGCGCGCC ctaacccatggcggtgacc 3′. HEK293T cells were cotransfected with any 2 combinations of the following plasmids: pcDNA3.1-RUNX3-FLAG, pcDNA3.1-GATA3-HA, and empty vector. After 48 hours, cells were harvested and lysed (cell lysis buffer, Cell Signaling Technology, catalog 9803). Cell lysates were incubated with mouse anti-FLAG magnetic beads overnight with gentle rocking in cold room (Sigma, catalog M8823); beads were washed several times using PBST (0.1% Triton X-100); and pellets were resuspended in 30 μL 3X or 6X SDS sample buffer, heated to 95–100°C for 5 minutes and spun down for Western blotting on 12%–15% SDS-PAGE gels. The following rabbit Abs were used: anti-HA, anti-RUNX3, and anti-GATA3 Ab (Cell Signaling Technology).

### ChIP-qPCR.

Human T cells were crosslinked in 1% formaldehyde for 10 minutes, followed by quenching with 0.125 M glycine for 5 minutes at room temperature. Chromatin was sheared using a Covaris sonicator (Covaris) to achieve a size between 100–300 bp. Sonicated chromatin solution was aliquoted and incubated with the anti-RUNX3 (BioLegend, catalog 653604) or anti-GATA3 Ab (Santa Cruz Biotechnology, catalog sc-268x) overnight at 4°C. Immunocomplexes were captured by protein-G magnetic beads (Life Technologies) followed by stringent washes and elution. The eluted samples were reverse cross-linked overnight at 65°C, and were then digested by RNase A at 37°C for 2 hours and proteinase K at 55°C for 30 minutes. DNA was purified by Qiagen QIAquick PCR purification kit and quantified using Qubit (Life Technologies).

qPCR was performed with LightCycler 480 SYBR Green kit (Roche). The signals were calculated as a percentage of input. The following primers were used: RUNX3_CD39P_Site1_F: 5′ CTTGACGGTCTGGATGTGGT 3′; RUNX3_CD39P_Site1_R: 5′ AGGAGCTGCTCAATGGGAAC 3′ and RUNX3_CD39P_Site2_F: 5′ GTTCCCATTGAGCAGCTCCT 3′; RUNX3_CD39P_Site2_R: 5′ AGAGCAACAGGTGACCCAAG 3′. GATA3_CD39P_Site1_F: 5′ TCAGCCACTCTGGATCTTCC 3′; GATA3_CD39P_Site1_R: 5′ TGAAAAGAGCATGTCCACAAA 3′; GATA3_CD39P_Site2_F: 5′ AAAATAATTTGGTCAGGCTCTCA 3′; GATA3_CD39P_Site2_R: 5′ GAGCATGAATTACCACATCCAG 3′; IL4P_F: 5′ GGAAACACACGGCTGAGAAT 3′; and IL4P_R: 5′ AACACACAGGGCTTCACTCC 3′.

### Luciferase reporter assay.

The *ENTPD1* promoter sequence (–1500 to +200) was inserted into the pGL3 basic plasmid. HEK293T cells were cotransfected with luciferase reporter plasmid, thymidine kinase promoter-Renilla luciferase reporter plasmid, a GATA3-expressing plasmid, or a control vector. After 48 hours, luciferase activities were determined by the Dual-Luciferase Reporter Assay System (Promega) according to the manufacturer’s instructions.

### Mice, LCMV infection, and AS1517499 treatment.

C57BL/6J (B6) mice were purchased from the Jackson Laboratory. LCMV-Armstrong was grown in BHK cells and titered in Vero cells. Naive CD4^+^ cells were isolated from CD45.1 SMARTA mice and injected into CD45.2 B6 WT mice through the tail vein. After 2 days, mice were infected with LCMV Armstrong at a dose of 2 × 10^5^ plaque-forming units, followed by daily i.p. injection of 10mg/kg STAT6 inhibitor AS1517499 (AXON Medchem, catalog Axon 1992) or DMSO solvent control. Mice were sacrificed after 1 week and CD39 expression on splenic SMARTA cells was determined. All mice were housed in the Stanford Research Animal Facility according to Stanford University guidelines.

### Characterization of TILs for CD39 and IL-4R expression.

C57BL/6J mice were reconstituted with OT-I cells via tail vein injection. After 1 day, mice were implanted with B16-OVA cells in the right flank. Tumor growth was monitored 2 times a week using digital calibers. Animal studies were performed with approval by the Mayo Clinic Institutional Animal Care and Use Committee.

Spleen and tumor were harvested on day 26. To analyze tumor-infiltrating T cells, tumors were cut into small pieces with scissors and digested for 30 minutes at 37°C in RPMI1640 media containing 500 μg/mL Collagenase type IA (Millipore Sigma) and 50 U/mL DNase I (Worthington Biochemical). Tumor cell suspension and splenocytes were washed with 2% FBS in HBSS (Thermo Fisher Scientific) and filtered through 70 μm cell strainers. After depleting red blood cells with ACK Lysing Buffer (Thermo Fisher Scientific), cells were treated with FcR-blocking reagent (BD Biosciences) and stained with Fixable Viability Dye eFluor 780 (Thermo Fisher Scientific), followed by cell surface staining with the following Abs: anti-CD8 Ab (53-6.7, BioLegend, catalog 100749); anti-CD39 Ab (Duha59, BioLegend, catalog 143805); anti-TIM3 Ab (RMT3-23, BioLegend, catalog 119727); and anti-IL-4RA (I015F8, BioLegend, catalog 144803). Mouse CXCR5 was stained with biotin-conjugated anti-CXCR5 (2G8, BD Biosciences, catalog 551960) binding to streptavidin BV421 (BioLegend). Cells were analyzed on a ZE5 (Bio Rad).

### RNA isolation and qRT-PCR.

Total RNA was isolated using either RNeasy Plus Mini or Micro kit (QIAGEN, catalog 74134 or 74034) depending on the cell number and converted to cDNA using High-Capacity cDNA Reverse Transcription Kit (Thermo Fisher Scientific, catalog 4368813). qRT-PCR was performed on an Eppendorf Thermal Cycler using Powerup SYBR Green Master Mix (Thermo Fisher Scientific, catalog A25776) according to the manufacturer’s instructions. Expression levels were normalized to *ACTB* expression and displayed as 2^–ΔCt^ × 10^5^. Primer sequences of CD39 and STAT6 were as follows: *ENTPD1*_QPCR_F: 5′ AGGTGCCTATGGCTGGATTAC 3′; *ENTPD1_*QPCR_R: 5′ CCAAAGCTCCAAAGaGTTTCCT 3′. *STAT6*_QPCR_F: 5′ GTTCCGCCACTTGCCAATG 3′; and *STAT6_*QPCR_R: 5′ TGGATCTCCCCTACTCGGTG 3′.

### Statistics.

Statistical analysis was performed using Prism (GraphPad). Paired or unpaired 2-tailed Student’s *t* tests were used for comparing 2 groups, and 1-way ANOVA with Tukey’s post hoc test was used for multigroup comparisons. *P* < 0.05 was considered statistically significant.

### Study approval.

Human studies were approved by the Stanford and the Mayo Clinic IRBs. Animal experiments were approved by the Stanford University Institutional Animal Care and Use Committee.

## Author contributions

FF, WC, YM, CMW, and JJG designed the study. FF, WC, YM, HO, LL, and JQ performed the experiments. WC, FF, WC, YM, CMW, and JJG analyzed and interpreted the data. FF and JJG wrote the manuscript with all authors providing feedback. FF, WC, and YM contributed equally to this study. FF initiated this study and therefore is listed first.

## Supplementary Material

Supplemental data

## Version 1 06/22/2022

Electronic publication
