# Supplementary material for: IL-4 prevents adenosine-mediated immunoregulation by inhibiting CD39 expression
Source: JCI Insight. 2022 Jun 22;7(12):e157509. doi: 10.1172/jci.insight.157509 (PMC9309057; doi:10.1172/jci.insight.157509)
Supplement: Supplemental data [file jciinsight-7-157509-s223.pdf]

Supplemental Material

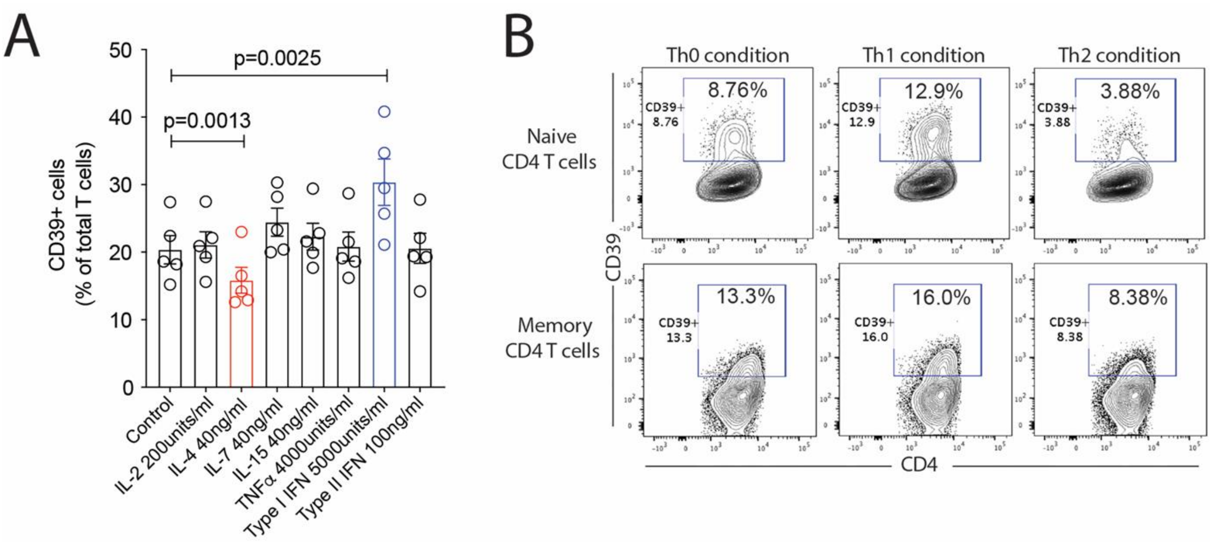

Supplemental Figure 1. IL-4 signaling prevents induction of CD39 expression on activated T cells. (A) Purified naïve and memory CD4 T cells were activated and differentiated under Th0 (TCR signaling only), Th1 (TCR signaling plus IL-12 and anti-IL-4 Ab) or Th2 (TCR signaling plus IL-4 and anti-IFNγ Ab) condition. **(B)** Purified total T cells were activated by anti-CD3/CD28 Dynabeads in the presence of indicated cytokines. 5 days later, CD39 cell surface expression was analyzed by flow cytometry. Data were compared by one-way ANOVA with Tukey's post hoc test.

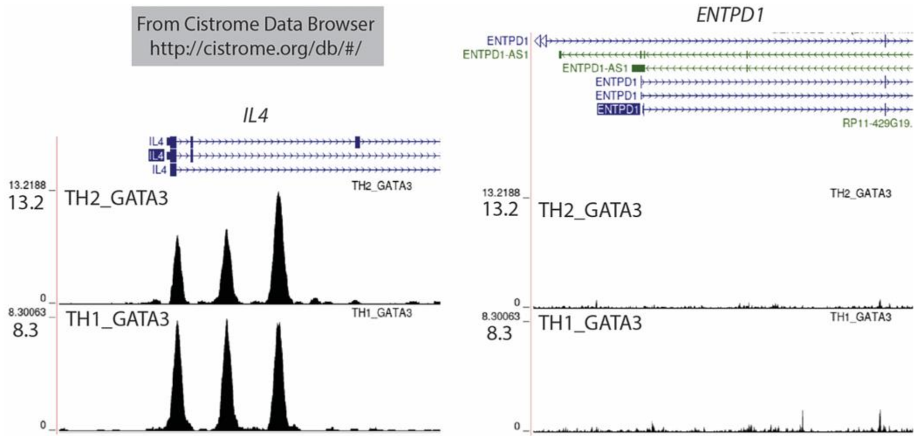

Supplemental Figure 2. GATA3 is not recruited to the *ENTPD1* promoter.
GATA3 CHIP-seq data from in-vitro differentiated human TH1 and TH2 cells were downloaded from Cistrome (Kanhere et al., 2012). Left panel shows GATA3 binding to the *IL4* gene; right panel shows the promoter region of *ENTPD1*.

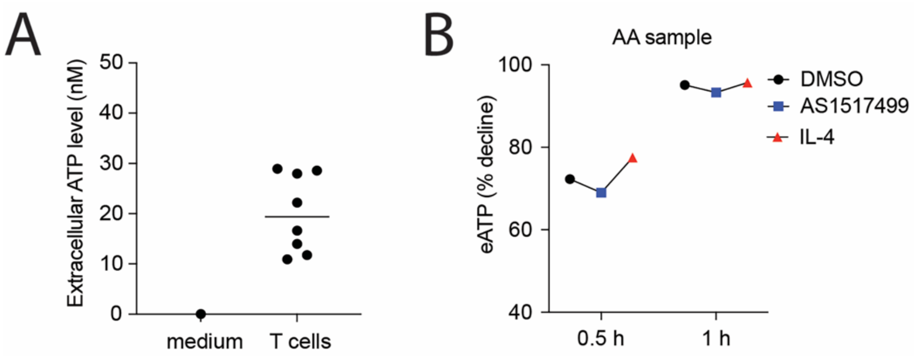

Supplementary Figure 3. Effect of CD39 expression on degradation of extracellular ATP (eATP). A. eATP concentrations were determined in medium and in supernatants of T cells activated for 4 days.

B. T cells from an individual with the CD39 low-expressor A/A SNP were activated in the presence of DMSO control, AS1517499 or IL-4. On day 4, cells were resuspended in medium supplemental with 20 µM ATP. eATP was determined after 30 minutes and one hour. Results are given as the percent decline from the original concentration.
